# Comparison Enhances Size Sensitivity: Neural Correlates of Outcome Magnitude Processing

**DOI:** 10.1371/journal.pone.0071186

**Published:** 2013-08-12

**Authors:** Qiuling Luo, Chen Qu

**Affiliations:** Center for the Study of Applied Psychology, South China Normal University, Guangzhou, China; Centre national de la recherche scientifique, France

## Abstract

Magnitude is a critical feature of outcomes. In the present study, two event-related potential (ERP) experiments were implemented to explore the neural substrates of outcome magnitude processing. In Experiment 1, we used an adapted gambling paradigm where physical area symbols were set to represent potential relative outcome magnitudes in order to exclude the possibility that the participants would be ignorant of the magnitudes. The context was manipulated as total monetary amount: ¥4 and ¥40. In these two contexts, the relative outcome magnitudes were ¥1 versus ¥3, and ¥10 versus ¥30, respectively. Experiment 2, which provided two area symbols with similar outcome magnitudes, was conducted to exclude the possible interpretation of physical area symbol for magnitude effect of feedback–related negativity (FRN) in Experiment 1. Our results showed that FRN responded to the relative outcome magnitude but not to the context or area symbol, with larger amplitudes for relatively small outcomes. A larger FRN effect (the difference between losses and wins) was found for relatively large outcomes than relatively small outcomes. Relatively large outcomes evoked greater positive ERP waves (P300) than relatively small outcomes. Furthermore, relatively large outcomes in a high amount context elicited a larger P300 than those in a low amount context. The current study indicated that FRN is sensitive to variations in magnitude. Moreover, relative magnitude was integrated in both the early and late stages of feedback processing, while the monetary amount context was processed only in the late stage of feedback processing.

## Introduction

Learning the association between behavioral response and their consequences plays a key role in enabling individuals to flexibly adapt to various environmental demands [1], [2], [3], [4]. Apart from valence, outcome magnitude, namely, the degree of goodness or badness associated with an outcome, is also important, and has been previously investigated [5], [6], [7], [8]. Stimuli such as stock quotation boards and performance reports can affect people’s nerves in light of their implications regarding economic costs and benefits. Larger magnitudes are often associated with stronger arousal and emotional feelings [2]. More pleasant feelings are associated with a big win, while negative feelings are associated with a big loss. Several prior studies have found that the magnitude of outcome also affect one’s behavior preference in decision-making [9], [10], [11], [12]. Markowitz (1952) offered two choices with different sizes of profits and losses. Participants were found to make safe choices when the value at stake was big, and risky choices when the value at stake was small. The so-called outcome magnitude effect leaves the question of whether the mechanisms underlying outcome magnitude and valence processing are identical.

A window to the neural basis of the outcome evaluation system was provided by the discovery of a component of the event-related brain potential, termed feedback–related negativity (FRN) [13], [14]. FRN refers to a negative deflection at frontocentral recording sites that peaks approximately 200 to 300 ms after feedback presentation, and whose amplitude is typically more pronounced for feedback stimuli associated with unfavorable outcomes than for those associated with positive outcomes [15], [16], [17]. However, outcome magnitude, another part of outcome evaluation in addition to valence, has not been confirmed to map to FRN.

The reinforcement learning theory of error-related negativity (RL-ERN theory) holds that FRN reflects the impact of phasic decreases in dopamine signals from basal ganglia on motor-related areas of the anterior cingulate cortex (ACC), suggesting that reward size is indexed by the FRN [7], [16], [18]. The sensitivity of brain activity to outcome magnitude is corroborated by results of neuroimaging studies showing increased ACC activation with larger outcomes [19], [20]. Nevertheless, several ERP (event-related potential) findings with classical experimental paradigms have not reached consensus on the sensitivity of FRN to outcome magnitude [13], [16], [21], [22].

In most of these studies, participants were asked to choose between cards that were unpredictably associated with monetary gains or losses of various magnitudes. Cues about the amount of monetary reward (e.g., “5” or “25”) were always presented on the screen before the decision was made. After that, the color of the chosen digit (e.g., green or red) showed the potential win or loss of the current trial. Results showed that the sizes of FRN for results of different magnitudes were not divergent. Therefore, it is suggested that FRN reflects the cognitive processes of performance evaluation or detection of prediction errors in a rough binary way, without reflecting the degree of deviation from expectancy. However, in these studies, the iconic color stimuli that indicated valence only may have made participants ignore the magnitude information. Here, one could expect participants would concern the magnitude more if symbols for outcome magnitude were emphasized and more accessible.

The present study attempts to use an adapted gambling paradigm to explore neural substrates of outcome magnitude processing in different contexts. Experiment 1 employed two symbols of physical area to represent potential relative outcome magnitude in order to exclude the possible ignorance of magnitude. The dramatic size difference of these two symbols was to remind participants of the magnitude involved in the current trial. Two different magnitude contexts were manipulated as ¥4 and ¥40. In the context of ¥4, the magnitude of gain and loss was ¥1 or ¥3, and in the context of ¥40, the magnitude was ¥10 or ¥30. In other words, 4 versus 40 served as the total monetary amount of context, while 1 versus 3 or 10 versus 30 served as the relative magnitude of outcome. We anticipated that FRN is sensitive to relative outcome magnitude when symbols for magnitude were more accessible. Moreover, if FRN only differed between relatively small and large outcomes, neural correlates of outcome magnitude could be evaluated in a context-independent fashion. Conversely, if FRN also differed between the ¥4 and ¥40 contexts, neural correlates of outcome magnitude could be evaluated in a context-dependent fashion. Experiment 2, which provided two different area symbols with the same outcome magnitude, was conducted to exclude the possible effects of physical area symbols on FRN in Experiment 1. If a similar FRN effect was observed for different area symbols, we could conclude that the physical area symbol should not affect the FRN amplitude.

## Experiment 1

### Methods

#### Participants

Eighteen undergraduate students (10 female; mean age 20.78±1.26 years) free of neurological deficits and psychiatric disorders participated in Experiment 1 as paid volunteers. All of the participants were right-handed, had normal or corrected-to-normal visual acuity, and reported normal color vision. They were informed that they would receive ¥15 for their participation, and that their performance would determine how much they would be awarded or penalized in addition to this basic payment. All participants gave written informed consent, and the study was approved by the Academic Committee of the Department of Psychology at South China Normal University.

#### Experimental procedure

All participants were seated comfortably in a dimly illuminated, acoustically and electrically shielded room. Stimuli were presented at the center of a black monitor placed at eye level 100 cm in front of them. The experiment used a 2 (context: low amount (¥4) context versus high amount (¥40) context) × 2 (relative outcome magnitude: small versus large (i.e., 1/4 vs. 3/4)) × 2 (valence: win vs. loss) factorial design, with the outcome being winning or losing ¥1 or ¥3 in a low amount context and ¥10 or ¥30 in a high amount context.

**Figure 1:**
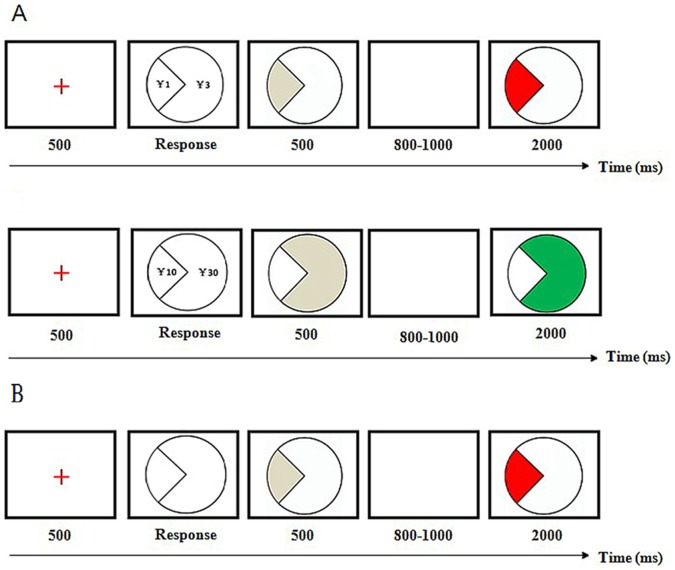
Sequence of stimuli in a typical trial. Time (ms) represents stimulus duration. (A) Tasks in low amount (¥4) context (upper panel) and in high amount (¥40) context (lower panel) in Experiment 1; (B) Task in Experiment 2.

We utilized a monetary gambling task adapted from that of Gehring and Willoughby (2002). Each trial began with the presentation of a red fixation cross for 500 ms, and the subsequent display of a round object (3° visual angle) with two parts. The area of the right part was triple that of the left, implicating a potential monetary magnitude difference between the right and left choice. In the ¥4 context, the digits of “1” and “3” were presented in the corresponding parts of the round object. Analogously, “10” and “30” appeared in the ¥40 context. During this choosing period, participants were informed that one card represented “winning” and another represented “losing”, and his/her task was to choose the winning card using whatever strategies he/she could. The participant selected one of two choices by pressing the corresponding response button with their left or right index finger. The part of the round object that the subject chose was highlighted by a padding of grey after a response period of 500 ms. Then, a blank screen was shown for a duration between 800 and 1000 ms. Finally, the grey color transformed into red or green, which informed the participant of the result. The color of the feedback display emphasized the utilitarian value of the feedback. For half of the participants, the green color in the feedback display emphasized a positive chosen outcome, and the red color emphasized a negative chosen outcome. For the other half of the participants, this assignment was reversed. The feedback stimulus that was displayed remained visible for a fixed duration of 2000 ms, followed by an intertrial interval of 1000 ms (Fig. 1A).

Before the ERP recordings, participants underwent a training session to become acquainted with the procedures. The formal experiment consisted of 4 blocks of 90 trials per block, with 2 blocks for every type of context. The numerals indicating outcome magnitude were presented throughout the entire trial during the training session, but only appeared in the choosing phase of the experimental session. At the beginning of each block, a picture of ¥4 or ¥40 would appear on the screen for 5000 ms, indicating the block type that would follow. At the beginning of the experiment, participants were informed that the value of five randomly selected trials would be added to (or subtracted from) the total amount of bonus money awarded to them at the end of the 4 blocks of trials. They were also told to earn as much money as possible using any strategy possible. Unbeknownst to the subjects, feedback was provided according to a pre-specified pseudorandom sequence with half the times winning and another half losing.

After the electroencephalography (EEG) session, participants were required to estimate the pleasure they derived from the eight types of outcomes they experienced using the 9-point Likert scale.

#### EEG recording and processing

Brain electrical activity was recorded at 32 scalp sites using tin electrodes mounted in an elastic cap (Brain products, Munich, Germany), with an online reference to the left mastoid and off-line algebraic re-reference to the average of the left and right mastoids. A ground electrode was placed on the medial frontal aspect. The vertical electrooculograms (VEOGs) were recorded from the left supraorbital and infraorbital regions. The horizontal electrooculogram (HEOG) was recorded by electrodes placed 1.5 cm lateral to the left and right external canthi. All electrode recordings were referenced to an electrode placed on the left mastoid muscle, and the inter-electrode impedance was maintained below 5 kΩ. The EEG and EOG were amplified using a 0.01- to 100-Hz band pass filter and continuously digitized at 500 Hz/channel for offline analysis. Ocular artifacts were identified and corrected with an eye-movement-correction algorithm. All trials in which EEG voltages exceeded a threshold of ±100 mV during the recording epoch were excluded from the analysis. EEG data were low-pass filtered at 30 Hz and were re-referenced offline to linked mastoid electrodes by subtracting one-half of the activity recorded at the right mastoid from each sample of data recorded at each channel. The data were baseline corrected by subtracting the average activity of that channel during the baseline period from each sample.

During the offline analysis, ocular artifacts were corrected with an eye-movement correction algorithm which employs a regression analysis in combination with artifact averaging [23]. Separate EEG epochs of 1000 ms (200 ms at baseline) were extracted offline for the feedback stimuli for each electrode. The analyzed ERP components included FRN and P300. For the purpose of statistical analysis, FRN amplitude was quantified as the average amplitude of the waveform 200–250 ms post-onset of feedback. The P300 for different results and electrodes was defined as the average amplitude of the waveform 300 to 400 ms after the onset of visual feedback. We focused on the four electrode locations in the midline (Fz, FCz, Cz and Pz), where these components had been most pronounced in previous studies. Analysis of variance (ANOVA) was conducted with four within-participant factors: electrode location, context, relative magnitude and valance. In all analyses, the Greenhouse–Geisser correction for nonsphericity was applied where appropriate.

### Results

#### Behavioral measures

In order to examine whether the preceding result affected the next action taken by each participant, we calculated the proportions of choices made by a given subject after the eight possible outcomes. A 2 (context) × 2 (relative outcome magnitude) × 2 (valence) × 2 (response type) repeated-measures ANOVA of choice distribution revealed a significant interaction between context and response type, F(1, 17) = 5.019, *p* = .039, suggesting a possible influence of outcome magnitude on behavioral choice. Further analysis demonstrated that participants tended to make more risky choices (that is, choices of ¥3 or ¥30) in the ¥4 (26.203±5.290%) than ¥40 context (24.608±4.244%), while more safer choices (that is, choices of ¥1 or ¥10) were made in the ¥40 (25.462±4.391%) than ¥4 context (23.727±5.208%), *p*<.05. A 2 (context) × 2 (relative outcome magnitude) × 2 (valence) repeated measure analysis of variance (ANOVA) conducted on the response time (RT) for making the initial choice in the trial following presentation of outcomes, yielded a marginally significant effect of valence factor, F(1, 17) = 4.456, *p* = .050, with a longer response time after wins (769.495±378.808 ms) than after losses (649.482±227.666 ms).

**Figure 2:**
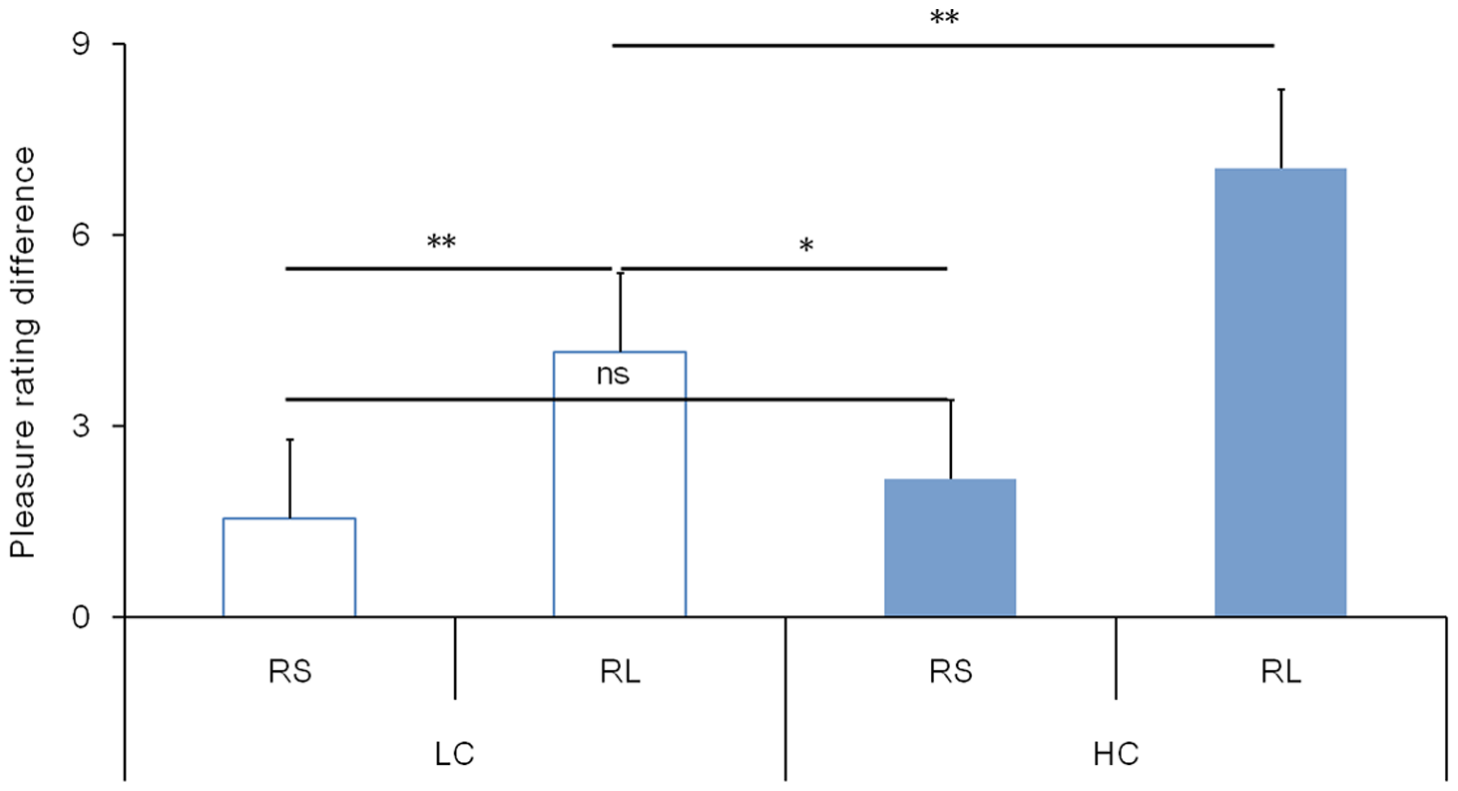
Difference in subjective rating of pleasure between losses and wins for results of different magnitudes. Error bars represent standard error (SE). RS, relatively small; RL, relatively large; LC, low amount context; HC, high amount context; *, significant difference of p<.05; **, significant difference of p<.005; ns, non-significant.

For self-reported pleasure, three factors repeated measures ANOVA with context, relative magnitude and valence as independent factors revealed significant effects of relative magnitude (F(1, 17) = 8.596, *p* = .009) and valence (F(1, 17) = 128.716, *p*<.001). Relatively large results were related to more pleasure than relatively small results were, while wins were related to more pleasure than losses were. The interaction between these two factors was significant, F(1, 17) = 62.705, *p*<.001. Further analysis revealed that the valence effect of pleasure (pleasure difference between wins and losses) was larger for relatively large results (F(1, 17) = 142.15, *p*<.001) than that for relatively small results (F(1, 17) = 62.70, *p*<.001). The interaction between context and valence was also significant, F(1, 17) = 15.207, *p* = .001. *Least significant difference* (LSD) post-hoc tests of this interaction demonstrated a larger valence effect of pleasure in the ¥40 context (F(1, 17) = 198.50, *p*<.001) than in the ¥4 context (F(1, 17) = 38.89, *p*<.001). A significant interaction between the three factors was also found, F(1, 17) = 6.682, *p* = .019. As can be seen in Figure 2, a trend of larger valence effect with increasing outcome magnitude did not exist between contexts but within context. The subjective pleasure difference between wins and losses of these four sizes of results were entered into a paired t test. The valence effect was largest in the ¥30 condition and smallest in the ¥1 condition. Although there was a large discrepancy in value between ¥1 and ¥10, the difference in the valence effects of pleasure elicited for these two magnitude conditions was not significant. An intriguing finding was that ¥3 elicited a larger valence difference of pleasure than ¥10 (*p*<.05), even though the former contained a smaller economic value.

#### ERP results

**Figure 3:**
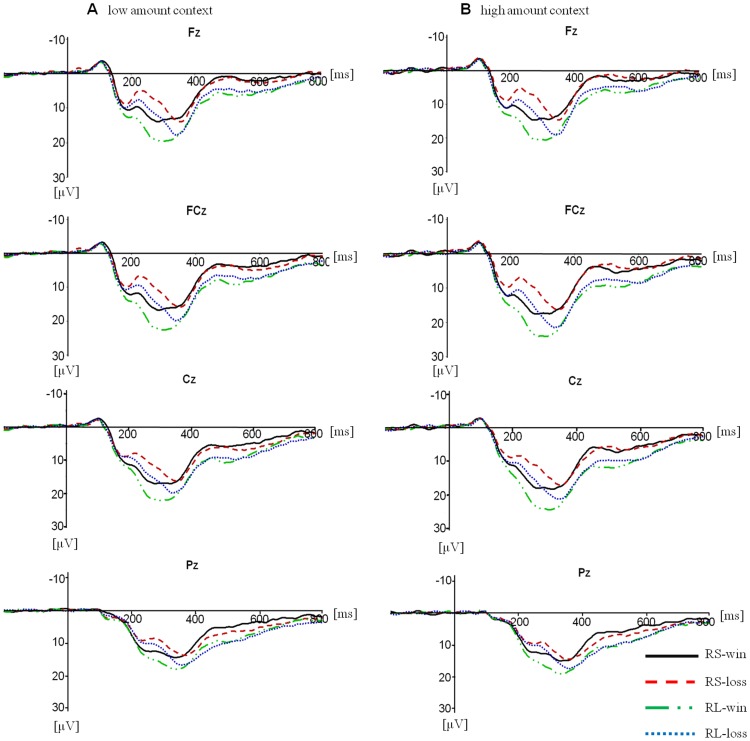
Grand average ERPs at Fz, FCz, Cz and Pz for four kinds of results in the low amount context (A) and in the high amount context (B). RS, relatively small; RL, relatively large.

**FRN.** Visual inspection of the grand average ERPs revealed a feedback related negativity beginning 200–250 ms post-stimulus onset (Fig. 3). The four factors repeated-measures ANOVA was conducted for the mean amplitude of FRN which yielded a significant main effect of electrode location, F(3, 51) = 5.717, *p* = .016. A pair-wise comparison confirmed that FRN amplitude was largest at Fz and smallest at Cz. The main effects of relative magnitude (F(1, 17) = 16.471, *p* = .001) and valence (F(1, 17) = 44.137, *p*<.001) were also significant, with more negative FRN for relatively small trials and loss trials than that for relatively large trials and win trials. A near-significant main effect of context was found, F(1, 17) = 3.620, *p* = .074. The mean amplitude of FRN for the low amount context was little larger than that for the high amount context, indicating that context has a similar but smaller effect on FRN amplitude than relative magnitude.

The relative magnitude factor interacted with the electrode location. The relative magnitude effect was different at four electrodes, F(3, 51) = 12.835, *p* = .002, with this effect significant at FCz, F(1, 17) = 16.26, *p* = .001, Cz, F(1, 17) = 22.23, *p*<0.001 and Pz, F(1, 17) = 13.55, *p* = .002, near-significant at Fz, F(1, 17) = 3.83, *p* = .067. The interaction of electrode and valence was extremely significant, F(3, 51) = 23.904, *p*<.001. Further analysis indicated that the FRN effect (that is, the amplitude difference between losses and wins), significant at all four electrode, was little larger at Fz (F(1, 17) = 50.96, *p*<.001) and FCz (F(1, 17) = 49.61, *p*<.001) than that at Cz (F(1, 17) = 39.61, *p*<.001) and Pz (F(1, 17) = 23.93, *p*<.001). A significant interaction effect between relative outcome magnitude and valence was found, F(1, 17) = 5.300, *p* = .034, indicating a little larger FRN effect for relatively large results (F(1, 17) = 47.32, *p*<.001) than that for relatively small results (F(1, 17) = 32.92, *p<*.001).

#### P300

The repeated-measures ANOVA for the average amplitude of all trials revealed a significant main effect of electrode location, F(3, 51) = 13.872, *p*<.001, suggesting a more positive P300 at Cz and FCz than that at Pz and Fz, *p*<.05. The main effect of context failed to reach significance, F(1, 17) = 3.653, *p* = .073, while the main effect of relative magnitude was significant, F(1, 17) = 21.740, *p*<0.001, with more positive P300 related to relatively large magnitudes. Moreover, this effect interacted with the context factor, F(1, 17) = 4.575, *p* = .047. Further analysis indicated that the relatively small outcomes elicited comparative P300 in the low and high amount contexts, *p* = .073, while the relatively large outcomes aroused divergent P300 between the two contexts (*p* = .019). The interaction of electrode location and valence was near significant, F(3, 51) = 3.185, *p* = .054, with an increasingly greater effect on P300 at more posterior sites.

### Discussion

This experiment investigated whether neural mechanisms of outcome processing are modulated by outcome magnitude over context. Participants had to increase their own budget by choosing one of two area symbols containing different monetary gains and losses. Behavioral results revealed a preference for choices of relatively large magnitude in the low amount context, and a preference for choices of relatively small magnitude in the high amount context. Such an outcome magnitude effect demonstrated the possible effect of outcome magnitude on risk preference during decision-making [9], [12].

The self-reported pleasure difference between wins and losses was also modulated by outcome magnitude. Participants experienced larger differences in emotion between wins and losses for relatively large results regardless of the ¥4 or ¥ 40 context. These behavioral findings proved that subjects did actually notice the outcome magnitude information in current study and were influenced by it during gambling. These results also corroborated the effectiveness of the experimental manipulation in our study for investigating the neural correlates of outcome magnitude evaluation.

As expected, FRN was sensitive to valence, with larger amplitudes for losses than for wins. Moreover, FRN responded to relative magnitude, with larger amplitudes for relatively small results than for relatively large results. This relative magnitude effect interacted with valence. A larger FRN effect was found with relatively large results than with relatively small results. P300 was found to be larger for relatively large results compared to relatively small results. The context interacted with relative magnitude in the component of P300. Relatively large results elicited larger P300 amplitudes in the high amount context than in the low amount context. Therefore, the difference between relatively small and large results can be indexed by FRN and P300. In contrast, the outcomes in different contexts tended to elicit diverse amplitudes only on P300 with an interaction with relative magnitude.

The main results of FRN indicated that the evaluation process of magnitude operates in a complex manner. First, FRN showed different susceptibilities to relative magnitude and monetary amount context, with stronger detection for the information of relative magnitude. Second, the processes of evaluating relative outcome magnitude interacted with that of valence evaluation, while the context evaluation did not.

However, there is an alternative account of outcome magnitude effect on FRN that reflects the possible mediation from area symbol. By virtue of the physical area difference, results with different sizes yield different patterns of FRN. Such an account seems likely given that perceptual conflict can be detected by the FRN in brain potentials [24]. Thus, Experiment 2 was necessary to ensure that relative outcome magnitude effects observed in feedback related ERPs were not solely due to physical area symbol.

## Experiment 2

Since physical area symbol might be a possible interpretation for FRN effect, we conducted Experiment 2 to tackle this problem. In this experiment, area symbol did not represent outcome magnitude. If a similar FRN effect was observed in different area symbols, that FRN effect was evaluated in an area-independent fashion. This would allow us to determine if the relative magnitude effect of FRN in Experiment 1 was not simply a reflection of physical area difference.

### Methods

#### Participants

Twenty two volunteers (eight men; mean age 20.364±1.6197 years) participated in Experiment 2. All participants were healthy and right-handed, had normal or corrected-to-normal visual acuity, and had normal color vision. None of them had a history of neurological or psychiatric disorders. Participants were informed that their performance in the task determined how much they would be awarded or penalized. All participants gave written, informed consent and were informed of their right to discontinue participation at any time. This study was approved by the Academic Committee of the Department of Psychology at South China Normal University.

#### Experiment paradigm

The experiment apparatus and procedure were the same as those for Experiment 1 with the following exceptions. The two parts of the round symbol did not signify different outcome magnitudes, both of which indicated ¥1. Moreover, the numerical indication of potential money was not presented in any phase of the Experiment (Fig. 1B). There were a total of 120 trials with a break during the task. The self-estimation of pleasure towards different results was also conducted when the experiment was finished.

#### EEG recording and processing

The parameter setting of ERP, the choosing of the components, and the defining of the component values were all the same as in Experiment 1. A repeated measured ANOVA was conducted with three within-participant factors: electrode location, area symbol, and valance. The Greenhouse–Geisser correction for non-sphericity was applied where appropriate in all analyses.

### Results

#### Behavioral results

The proportion of choosing large area symbol (54.32±7.75%) was significantly larger than that of the small area symbol (45.68±7.75%), *p* = .018. To examine whether the preceding result influenced the next action taken by each participant, we analyzed the response times (RTs) for making the initial choice in the trial following presentation of one of the four possible outcomes. Repeated measures ANOVA, using area symbol (large vs. small) and valence (win vs. loss) as independent factors, did not reveal any significant effects (*p*>0.1).

**Figure 4:**
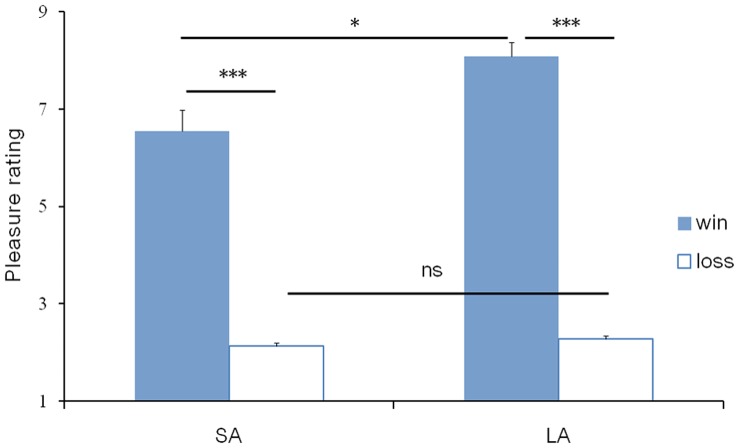
Subjective ratings of pleasure. The difference of pleasure between small and large area symbol results was larger in winning conditions than in lossing conditions. Error bars represent SE. *, significant difference at *p*<.05; ***, significant difference at p<.001; ns, non-significant; SA, small area symbol; LA, large area symbol.

The same analysis was conducted for the self-reported pleasure, and the results showed a very significant effect of area symbol, F(1, 21) = 10.820, *p* = .003. Further tests indicated that large area symbol trials elicited more positive emotions than small area symbol trials. The main effect of valence factor was also significant, F(1,19) = 117.902, *p*<.001, with more pleasant emotions for wins than for losses. Moreover, these two factors interacted with each other, F(1, 21) = 6.985, *p* = .015. Simple effect analysis found that the area symbol effect was notable in winning conditions (Fig. 4).

#### ERP results

**Figure 5:**
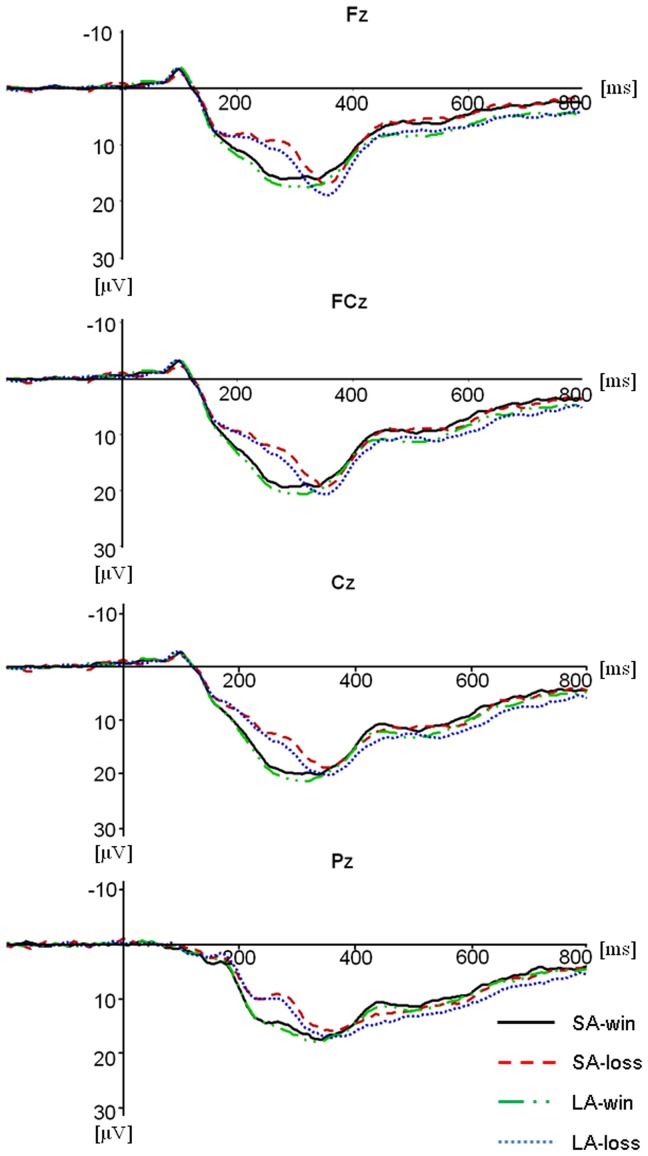
The grand average of ERPs at Fz, FCz, Cz and Pz for all four kinds of results. SA, small area symbol; LA, large area symbol.

**FRN.** Figure 5 depicts the grand average waveforms for feedback signals showing all combinations of area symbol and valence at Fz and FCz. A clear negativity in different conditions was observable with a maximum at Fz. Mean amplitudes in the 200–300 ms time window post-onset of feedback, defined through visual inspection, were entered into repeated measures ANOVA. There were significant effects of electrode location, F(3, 63) = 4.506, *p* = .034, and result valence, F(1, 21) = 27.839, *p*<.001. Further tests indicated that FRN amplitude was largest at FCz and smallest at Pz. There were more negative ERP responses for losses than wins. These two effects interacted with each other, F(3, 63) = 6.242, *p* = .012, with a significant valence effect at all four locations, but a larger effect at more anterior sites. Moreover, the interaction between electrode location and area symbol was significant, F(3, 63) = 5.163, *p* = .018. Post-host analysis suggested that the amplitudes of FRN for small and large area symbols were not significantly different at any site.

#### P300

Analysis of P300 amplitudes showed a significant effect of electrode, F(3, 63) = 11.074, *p*<.001. P300 was larger at more middle and posterior sites. The effect of area symbol factor was also significant, F(1, 21) = 9.439, *p* = .006, with a larger P300 for large area symbol condition than for small area symbol condition. This effect interacted with the electrode location, F(3, 63) = 3.364, *p* = .049. The area symbol effect was remarkable at all sites, but a little larger effect at Pz than others. Moreover, the electrode factor interacted with valence, F(3, 63) = 4.445, *p* = .032. Further tests indicated that valence effect was only significant at Pz.

### Discussion

Experiment 2 manipulated different area symbols with the same outcome magnitude to verify whether the FRN effect was modulated by the physical area perception. Results showed that participants were inclined to choose larger area symbols during the gambling. Moreover, participants felt more pleasure for large area symbol results than for small area symbol results, even though these two results signified equivalent ¥1. This area effect on emotion embodied the irrational side of decision-making.

The valence effect of FRN was observed in this experiment, which replicated previous findings. FRN associated with losses was more pronounced than that associated with wins [5], [16], [21], [22], [25]. Importantly, FRN was also not modulated by the area symbol. These findings ruled out the possible impact of area symbol on feedback evaluation and indicated the feasibility of manipulating area symbols to signify different outcome magnitudes. However, area symbol factor gradually had influence in the late phase of outcome evaluation. P300 showed dramatic sensitivity to area symbol, and large area symbol results embodied higher emotional arousal than small ones, although different area symbols possessed the same objective value. In the post-experiment interview, participants reported that they chose more often large area symbols because they believed it would bring them more luck. And yet after all that, it was unsuitable to trace the significant relative magnitude effect of the P300 findings in Experiment 1 to the physical area difference, because the area symbol effect of P300 in Experiment 2 was much smaller than the relative magnitude effect of P300 in Experiment 1.

## General Discussion

The present studies were implemented to explore the neural substrates of outcome magnitude effect. Experiment 1 employed a modified gambling task in which valence, context and relative outcome magnitude were manipulated. The context was defined as total monetary amount (e.g., ¥4 and ¥40). The relative outcome magnitude (e.g., ¥1 vs. ¥3 and ¥10 vs. ¥30) was implemented by two physical area symbols. Experiment 2 was conducted to exclude the possible interpretation of physical area symbol for FRN effect in Experiment 1.

As expected, FRN as one important component in feedback evaluation, was sensitive to valence. More importantly, the size of FRN was also modulated by the factor of relative outcome magnitude, with larger sizes for outcomes of relatively small magnitude than for that of relatively large magnitude. The results of Experiment 2 proved that this relative outcome magnitude effect was not affected by physical area symbol. Moreover, a larger FRN effect was found for relatively large magnitude than relatively small magnitude. As a late component that was closely connected with emotion and motivation, P300 was sensitive to relative magnitude. When it comes to the context factor, P300 could only discriminate between the difference of relatively large magnitude in the ¥4 and ¥40 contexts.

### Feedback Related Negativity and Outcome Magnitude

The current findings provide further insights into the psychological basis of outcome magnitude effect. First, discriminative sizes of FRN for different relative magnitude indicated that participants distinguished the relatively small results from large results during the early evaluation phase. Previous studies in which valence was represented by color and outcome magnitude was represented by digit cues found that FRN was sensitive to valence rather than outcome magnitude [5], [18], [21], [26]. We proposed that color as a salient symbol may facilitate valence processing resulting in less attention to outcome magnitude. The present study used an adapted paradigm in which, valence was represented by color and magnitude was represented by physical area symbol. This new way of presenting feedback helps to disentangle the possible ignorance of magnitude in previous research and making outcome magnitude more accessible. As expected, FRN was found to be modulated by relative magnitude, demonstrating that outcome magnitude was processed in the early stage of outcome processing.

A second intriguing finding in our research was the augmentation of the FRN effect (FRN amplitude difference between loss and win) for relatively large results. This FRN result pattern replicates pervious findings [7], [8], [27], which provides support for the RL theory. According to this theory, the FRN reflects the activity of a reinforcement learning system and codes the size of negative prediction error (difference between actual and expected outcomes) [17]. In current research, the value of the relatively large results was triple that of the relatively small results, which resulted in a larger prediction error for the former results. Therefore, distinct FRN effects for different relative outcome magnitudes may indicate that the magnitude modulates the amplitude of FRN by prediction error. Besides, the mutual influence between outcome valence and outcome magnitude may also reflect an impact of motivation/affection toward outcome evaluation [25], [28]. The larger the relative magnitude, the more emotion arousal or motivation was experienced, which may amplify the subjective feeling difference between wins and losses.

Third, context which determined the total monetary amount was also introduced to explore outcome evaluation. Results revealed that the context effect of FRN was far less than that of the relative magnitude effect. This discrepancy demonstrated that FRN is sensitive to the features of current outcomes and has a lessened capability to appraise feedback features cross situation. We have to admit that there is an alternative explanation. Participants’ attention is drawn to relative magnitude information by experimental manipulation in the current trial. This attention focus may have an effect on the FRN results. We could not completely rule out this possible influence. However, the behavioral findings proved that participants did notice the difference between ¥4 context and ¥40 context. Further investigation may use different paradigms to address this issue.

The finding of magnitude effect showed a special pattern that relatively small outcomes elicited a more negative FRN, which seems to contradict the RL theory of FRN. FRN, which was associated with unfavorable outcomes, such as incorrect responses or monetary loss, was more pronounced than that associated with favorable outcomes [4], [24], [29]. However, outcome magnitude can be either positive or negative. Relatively small outcomes could be negative in a winning condition where possible larger outcome could have been won, and yet, it could also be positive in a losing condition where larger possible amount could have been lost. The current larger FRN for relatively small magnitude than relatively large magnitude was in accordance with data from previous studies [5], [7], [8]. Only a few researchers tried to explain this FRN magnitude effect by expectation theory. They presumed that small results elicited a large FRN apart from expectation [8]. Participants were not always expecting a large result. Nevertheless small results would be preferable in a losing condition. In the current study, relative to small magnitude, participants choosing relatively large magnitude were more prepared to suffer from the predicted big loss (e.g., −3 or −30). More specifically, loss in large magnitude condition was closer to expectation than loss in small magnitude condition, which might cause a larger FRN for small magnitude and a smaller FRN for large magnitude. However, without further evidence, this idea is only a hypothesis. In any case, this puzzling “*value the small and slight the large”* pattern of FRN for relative magnitudes requires further exploration.

### Time Course of Outcome Magnitude Processing

Seen from the entire time interval of outcome evaluation FRN and P300 all showed sensitivity to the relative magnitude of results, while only P300 responded weakly to context which provided total monetary amount information of outcomes.

In outcome evaluation, P300 was chosen to comprehend the emotion processing of outcomes. This component has proved to be closely connected to emotion and motivation [5], [30], [31]. Previous studies found that comparing with neutral pictures disgusting ones elicited a prominent positive deflection [32], [33], [34]. In the current study, it is likely that relatively large outcomes represented stronger arousal and emotional feelings and were therefore associated with more positive P300 amplitudes. Moreover, the context information was encoded by P300 with an interaction with relative magnitude. The modulation of P300 amplitude by context was observed in relatively large magnitude trials, but not in relatively small magnitude trials. This segregation is due to a larger monetary difference between ¥4 and ¥40 contexts in the former case than that in the latter. This pattern of P300 results strongly suggests that later feedback processing stages serve to integrate relative magnitude and context information, thereby likely subserving decisional and evaluative processes.

These findings demonstrated the temporal course of processing relative magnitude information and monetary amount context information. In the early stage, FRN showed sensitivity to valence and relative magnitude, which had greater and immediate significance for our choice during gambling. In the late stage, the context information, which was indexed by P300, was integrated to make the best decision.

### Conclusions

With a more accessible symbol representing relative outcome magnitude, the present study demonstrates that the size of the FRN was modulated by the relative magnitude rather than monetary amount context, and the amplitude of the FRN in response to relatively small outcomes was much larger than relatively large outcomes. Such facilitated processing of relative magnitude is potentially beneficial in rapid decision-making. Furthermore, P300 showed discrete sensitivity to relative magnitude and context. This discrepancy is likely to reflect a possible segregation time interval for the neural processing of different types of outcome magnitude. Relative magnitude was integrated in both the early and late stage of feedback processing. However, the monetary amount context was only involved in the late stage with an interaction with relative magnitude.
